# Knowledge, attitudes and practices regarding HIV/AIDS among senior secondary school students in Fako Division, South West Region, Cameroon

**DOI:** 10.1186/s12889-016-3516-9

**Published:** 2016-08-22

**Authors:** Colins Kingoum Nubed, Jane-Francis Tatah Kihla Akoachere

**Affiliations:** Department of Microbiology and Parasitology, Faculty of Science, University of Buea, Buea, Cameroon

**Keywords:** Knowledge, Attitude, Practices, HIV/AIDS, Prevention, Control

## Abstract

**Background:**

Knowledge, attitudes and practices (KAPs) regarding HIV/AIDS is one of the corner stones in the fight against the disease. Youths are most vulnerable to infection because they engage in risky practices due to a lack of adequate information. Thus, evaluating their KAPs will help in designing appropriate prevention strategies. This study was aimed at assessing the KAPs of senior secondary school students in Fako Division, Cameroon, on HIV/AIDS.

**Methods:**

This was a cross-sectional study carried out on 464 students aged 13–25 years, selected by systematic quota random sampling from some secondary schools in Fako, from April to June 2014, to evaluate their KAPs regarding HIV/AIDS. Participants were drawn from one secondary school in each of the four health districts in Fako. Pre-tested questionnaires were administered to the students to obtain information about their KAPs on HIV/AIDS. Data were analyzed using SPSS version 20.0.

**Results:**

All respondents were aware of HIV/AIDS. Sources of information varied, the most common being sex education in school. The majority of participants demonstrated an adequate understanding of HIV transmission and prevention. However, misconceptions about routes of transmission were observed in 3.4 to 23.3 % of respondents. Risky behaviors were found among participants as about 60 % practice safe sex and 40 % reported not to. Up to 196 (42.2 %) respondents had a history of sexual intercourse of which 108 (56.25 %) had used a condom during their last three sexual encounters. About half of the respondents had negative views about HIV infected people. Students with medium (34.3 %) and high (62.1 %) levels of knowledge were more likely to display positive attitudes Although statistically not significant, we found that as knowledge increased the ability of respondents to report safer sex decreased (95 % CI, *P* = 0.922).

**Conclusions:**

Students had a satisfactory level of knowledge on HIV/AIDS prevention. Those with adequate knowledge were more likely to display positive attitudes towards PLHIV. Having adequate knowledge did not imply engaging in safe practices. This study none-the-less highlighted some misconceptions about HIV transmission, intolerant and discriminatory attitudes towards PLHIV, and risky sexual practices among study participants which can be corrected by reinforcing sex education curriculum as sex education in school was their main source of information on HIV/AIDS.

**Electronic supplementary material:**

The online version of this article (doi:10.1186/s12889-016-3516-9) contains supplementary material, which is available to authorized users.

## Background

Since 1981 when the first cases of AIDS were reported in the United States, HIV/AIDS infection has spread rapidly to many countries over the years and became a global health challenge. The disease continues to affect millions of people irrespective of age or sex. Estimates show that globally at the end of 2013, 35 million (33.2–37.2 million) people were living with the infection and 1.5 million deaths were recorded due the diseases, [1]. Although global statistics reveal a general decline in AIDS related deaths and new HIV infections thanks to the concerted efforts of various stakeholders, the toll of HIV/AIDS continues to be harsh in developing countries particularly those in sub-Saharan Africa. As of 2012, 71 % of people living with HIV worldwide were in sub-Saharan Africa which also accounted for 70 % of new infections and approximately 74 % of all deaths related to AIDS [2]. Worldwide, over 40 % of new infections are among young people 15–25 [3]. The youth are much more prone to HIV infection as well as other sexually transmitted infections as a result of a lack of correct health information, engagement in risky behaviors, economic exploitation, regional and national conflicts and a lack of access to adequate reproductive health services [4]. Every day 5000 young people in the world become infected with HIV, which translates into almost 2 million new infections per year [5].

In Cameroon the first cases of AIDS were reported in 1985. Since then, the disease escalated over the years to a prevalence of 11 % in 2000 [6]. Although recent reports are indicating a decline in prevalence from 5.1 % in 2010 [7] to 4.3 % in 2013 [8], mainly as a consequence of the implementation of the national strategic plans [9], in 2013, Cameroon was not listed among the countries that had met the 2011 UN Political Declaration on HIV and AIDS of reducing HIV transmission by 50 % [10]. Prevalence rates vary from one region of the country to another, and the South West region ranks 4^th^ [11]. HIV transmission in Cameroon is primarily heterosexual, and women are more vulnerable, with 170 infected women for every 100 infected men [12].

In 2013, 600,000 (560,000–650,000) people in Cameroon were estimated to be living with HIV and AIDS [8]. The youth are among the population at risk for contracting the infection as they engage in unsafe practices [13, 14]. The prevalence is highest in the 15–24 year age group with females being more affected than males [9]. Being that the youth are the future leaders and work force of the country, interventions targeted at the young people before they are sexually active could prevent new HIV infections and enable Cameroon to achieve its AIDS targets.

Knowledge, attitudes and practices (KAPs) regarding HIV/AIDS is one of the corner stones in the fight against the disease. Adequate knowledge about HIV/AIDS is a powerful means of promoting positive attitudes and engaging in safe practices. Many prevention programmes have focused on increasing knowledge on transmission so as to overcome misconceptions that could prevent behavioral change towards safe practices [13] and also reduce the stigma against people living with HIV/AIDS. Stigmatizing attitudes have been shown to be strongly associated with misconceptions on HIV transmission and are negative attitudes towards people living with HIV [15]. An assessment of KAPs among any population is highly necessary in planning the management and prevention of HIV, and as baseline to evaluate the success of prevention strategies. Studies involving the youth [16, 17] carried out in other divisions of Cameroon have documented a high level of awareness of HIV/AIDS but knowledge on various specific aspects relating to HIV/AIDS remain poor, with high levels of risky behaviors such as having multiple sex partners and inconsistent use of condom [18–20]. Despite their engagement in risky behaviors the majority of youths do not perceive themselves to be at risk of contracting the infection [21]. Still, other studies [22] have documented positive changes in condom usage among youth in two major cities in Cameroon, as a consequence of a youth focused intervention program.

In Cameroon like in many developing countries, HIV prevalence is higher in urban than in rural areas with the youth contributing to the high prevalence. Information on KAPs regarding HIV is scarce in Fako Division, despite the fact that it is the most urbanized and cosmopolitan division in the South West region of Cameroon, with an increasing youth population due to increased job opportunities and educational institutions. Studies involving youths in some parts of Fako have focused on other aspects relating to HIV. Haddison et al. [22] investigated the utilization of voluntary counseling and testing among high school students in Tiko. Tarkang [23] reported high risky behaviors among female high school students with early sexual debut in Limbe. Perceived barriers to condom use, perceived condom use, self-efficacy and socio-demographic variables were the most important correlates of consistent condom use in this study population [24]. In Buea, Nkuo-Akenji et al*.* [25] reported on knowledge of HIV/AIDS, sexual behavior and prevalence of sexually transmitted infections among female university students*.* We are not aware of any study involving KAP regarding HIV targeting secondary school students in Fako division. Information on KAP among secondary school students is important in designing intervention strategies to protect them from infection. This is very necessary and prepares them for the university when they leave home and are no longer under parental guidance, and may take wrong decisions on sex due to poor knowledge, increasing their risk of infection. This study was motivated by a desire to determine the level of understanding of youths in Fako division on HIV/AIDS, identify their behaviors which could pose a risk to infection with HIV and evaluate their attitudes towards people living with the disease.

## Methods

### Study site, study design and study period

This study was conducted in the Fako Division, South West Region of Cameroon. Fako Division is located at Latitude 4.1667° and Longitude 9.1667°. Of the six divisions in the South West region, Fako is the only one with several urban and semi-urban towns. It has many social and economic amenities and political institutions which in addition to its location in the coastal area of Cameroon have contributed to its increasing population, the majority of who are the youths. Fako Division has 4 health districts (HD) ; Tiko, Buea, Muyuka and Limbe.

This study was a cross-sectional descriptive study carried out from April to June 2014.

One secondary school was selected from each of the health districts in Fako. The names of schools in each health district were written on pieces of paper, mixed, and one was picked at random. From the selected schools respondents were recruited from senior classes: Form Five (11^th^ year of studies, on average 13–17 year-old, Lower and Upper Six) after they signed an informed consent form. Students were selected by systematic quota random sampling technique. Participation of the students was voluntary. All volunteers were moved into a separate hall. Participants were randomly selected from those who fell within our desired age subgroup. Questionnaires were pre-tested on students from schools not selected for this study. Previous studies [22, 24] reported age at sexual debut in students in Fako to be about 15 years. There is no existing data on KAPs among secondary school students in Fako. Pre-tested questionnaires were then administered to students in each of the selected schools to obtain information about their knowledge, attitudes and practices regarding HIV/AIDS. Students of both sexes were involved in the study and those who were less than 13 and above 25 years old were excluded. After the desired number of students was obtained we stopped distribution of questionnaires to volunteers.

Ethical clearance was obtained from the Institutional Review Board (IRB) of the Faculty of Health Sciences, University of Buea. Administrative clearance was obtained from the Regional Delegations of Public Health and Secondary Education. Written consent was obtained from participants above 21 years of age. For those below 21 years, a school staff/administrator signed their consent forms as legal guardian while they gave verbal assent to participate. Our point of contact was on school campuses during school hours and lecture periods. During this time only students were present on campuses thus asking their parents to be present during the survey or going to their various houses or job sites to obtained consent was not feasible considering the diversity of the student population and the different locations of their parents or legal guardians. Obtaining consent from the parents of each of these participants was not possible because the study was conducted when schools were about closing for the term and it would not have been possible to get all parents/guardians of the participants. In addition, because the participants were targeted at schools it was more feasible for members of the administrative body of each school (many of who were parents/guardians of students in those schools) to stand as legal guardians for informed consent purposes. The consideration at this point was that during school hours and lecture periods anything that happened or was happening to the students was under the control of the school administration and thus authorization to participate in the survey was obtained from the school administration after they went through the questionnaire to be administered to students. This was deemed appropriate by the Institutional Review Board and ethical clearance to conduct the study was granted. Information provided by the participants was anonymous and was kept confidential.

### Data collection technique

Pre-tested standard questionnaires with both open and close ended questions were administered to participants to obtain information on their level of KAPs on HIV/AIDS, as well as their sources of information on the issue. The questionnaires were designed based on the AIDS Indicator Survey model developed by the MEASURE DHS program and the AIDS survey model with some indicators from the National HIV/AIDS prevention programs for young people guide [25]. The final questionnaire included questions relating to HIV knowledge, attitudes toward people living with HIV (PLHIV) and sexual practices, in addition to socio-demographic information. The questionnaire was divided into four sections. Section I focused on the socio-demographic characteristics of the respondents. Section II contained determinant indicators (risk factors and protective factors) items which are knowledge-related items. These were subdivided into five sections, with questions relating to sources of information about HIV/AIDS, transmission, and prevention and management of HIV/AIDS. Mostly questions were used to assess their knowledge, as well as their misperceptions, about HIV/AIDS. Section III comprised questions on attitudes towards people living with HIV/AIDS (PLHIV), which required mostly “Yes/No” responses. Finally, Section IV comprised questions about sexual behavioral practices.

### Data analysis and interpretation

To evaluate knowledge and practices, respondents, were required to provide mostly “yes”, “no” responses and to elaborate in the allocated space where necessary. For attitude-related questions, options were only “yes” or “no”. A score of 1 was assigned for a correct answer and 0 for a wrong answer for the knowledge and practice-related questions that were strictly “Yes/No”. For the attitude questions, a score of 1 was assigned to a positive answer and 0 for negative answers. The scores were then summed up to obtain an overall score for each participant. Levels of knowledge were categorized into “low” for respondents who scored 50 % and below, “moderate” for those who scored between 51 and 74 %, and “high” for those who scored 75 % and above. The scores of attitudes and practices were categorized into ‘negative’ and ‘positive’ based on their mean score. Those scoring less than mean scores for attitude were classified as having ‘negative” attitudes and those scoring equal and more than mean scores were classified as having “positive” attitudes. With practice data not being normally distributed, the median was used as the cut-off point. Those scoring less than median scores were classified as “risky” practices, and those scoring equal and more than median scores were classified as “safe” practices.

Data were analyzed using Statistical Package for the Social Science® (SPSS), version 20.0 (SPSS Inc., Illinois, USA). Both qualitative and quantitative data were collected. Quantitative variables were summarized by median and interquartile range (IQR), or by mean and standard deviation (SD). For qualitative variables the number and percentage of subjects in each category were given. Bivariate correlation tests at 95 % confidence intervals (CIs) were calculated through a logistic correlation model to determine association between levels of knowledge with attitudes and practices. For demographic comparisons, the ANOVA test was done to determine if there was a variation of KAP between males and females of in the study population. The respondents were further grouped into three sub age groups, 13–16, 17–20, and 21–24 years. The ANOVA test was done to determine if KAP variations existed amongst these sub age groups in our study population. All tests were two tailed, and *P* < 0.05 was considered significant.

## Results

### Socio-demographic characteristics

The mean age of the participants was 17.62 years (SD ± 1.994), ranging from 13 to 24 years. Of the 464 respondents, 224 (48.3 %) were males and 240 (51.7 %) females. The majority were Christians. The distribution of the study participants is shown in Table 1.

**Table 1:** Socio-demographic characteristics of the study population

Characteristics	Number	%
Sex		
1. Male 2. Female	224 240	48.3 51.7
Religion		
1. Christianity 2. Islam	460 4	99.1 0.90
Age Mean ± SD = 17.62 ± 1.994 Range = 13 – 24 Years		

### Knowledge of HIV/AIDS transmission, prevention and control

**Fig. 1:**
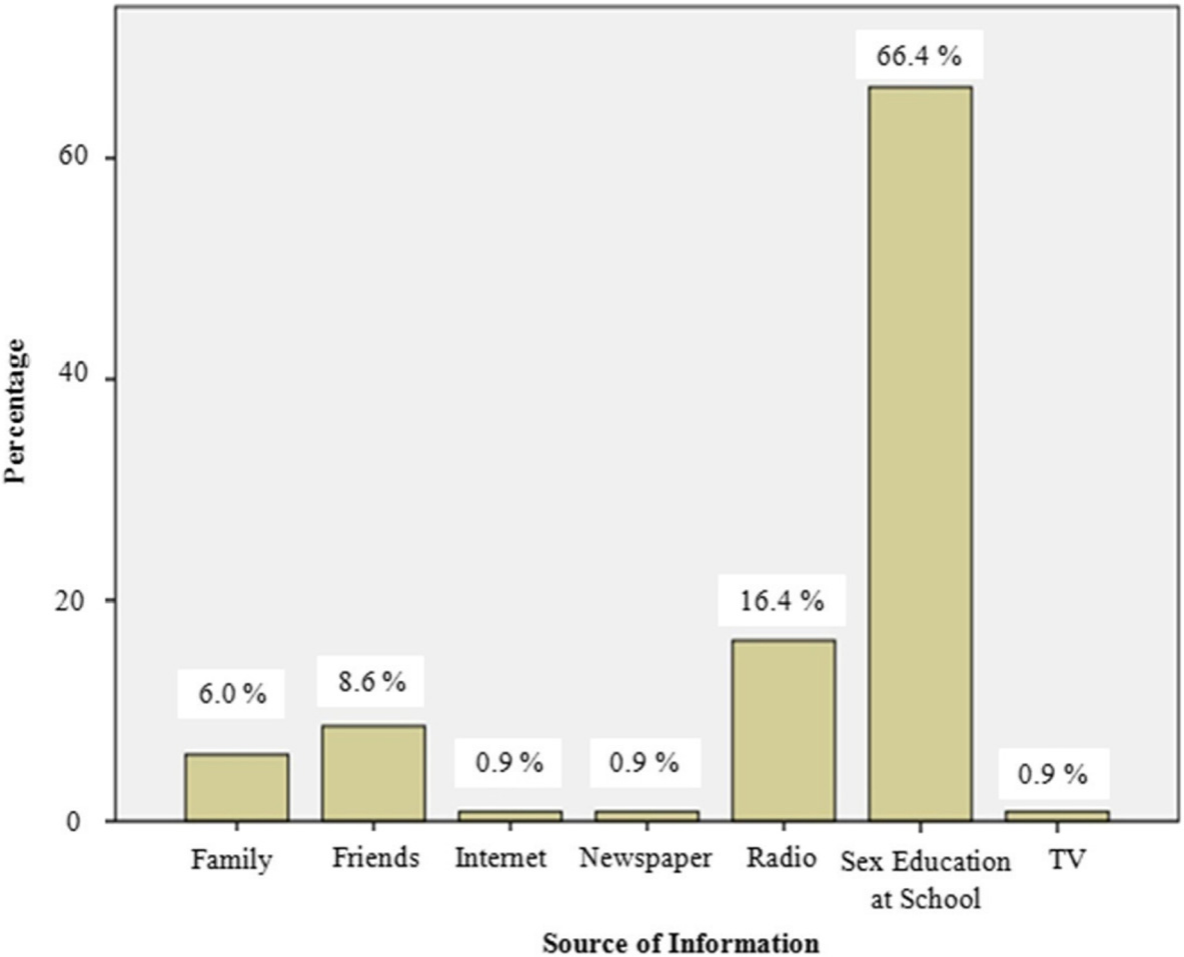
Source of information on HIV/AIDS reported by participants

All respondents had heard about HIV/AIDS but their sources of information varied. As illustrated in Fig. 1, 308 (66.4 %) obtained their information on HIV/AIDS from sex education at school, 76 (16.4 %) from the radio, 40 (8.6 %) from friends while 28 (6.0 %) heard about HIV from family members. Less prominent sources were the internet, newspapers and TV. The majority (98.8 %) (Table 2) acknowledged that a healthy looking person can have the infection.

**Table 2:** Knowledge on HIV/AIDS transmission, prevention and control

Question	Percentage with correct answer	Percentage reporting with wrong response (Misconceptions)
Knowledge on route of transmission		
1. Can a person get HIV infection from mosquito bites?	76.7	23.3 %
2. Can a person get HIV infection by sharing a meal with someone who is infected?	92.2	7.8 %
Knowledge on prevention and control		
3. Can the risk of HIV transmission be reduced by having sex with only one faithful uninfected partner?	84.5	15.5 %
4. Can a healthy-looking person have HIV infection?	98.8	1.2 %
5. Does condom use reduce risk of HIV transmission?	85.3	14.7 %
6. Can the risk of HIV transmission be reduced by abstaining from sexual intercourse?	90.5	9.5 %
7. Can an HIV-infected male be cured of HIV if he has sex with a young girl who is a virgin (a girl who has never had sex before)?	96.6	3.4 %
8. Is there a cure for HIV/AIDS?	81.9	18.1 %

For participants who selected more than one source, each one was considered during data entry.

To evaluate participants’ knowledge on routes of transmission of HIV/AIDS respondents were asked to list four routes of transmission. Correct responses included unprotected sexual intercourse, transfusion of infected blood and blood products, transmission from infected mother to child and sharing of infected blades, needles and unsterilized medical equipment. Those who listed all four correct were graded as “Excellent” knowledge, 3 correct as “Good”, 2 correct as “Fair” and only one or none correct as “Poor”. Only 30.2 % of our respondents had excellent knowledge of routes of transmission of HIV. The majority (48.3 %) had good knowledge. However, those with poor knowledge comprised only 3.4 % of the study population.

To further confirm the participants’ level of understanding of HIV transmission, they were asked more questions on routes of transmission (Table 2). Those with wrong responses had misconceptions. Up to 23.3 % thought that HIV could be transmitted by mosquito bites while 15.5 % thought having sex with only one uninfected faithful partner could result in transmission.

With regards to knowledge of prevention and control, 384 (82.2 %) indicated that transmission could be reduced by having sex with one faithful uninfected partner, 396 (85.3 %) indicated that the use of condoms could reduce the risk of HIV/AIDS transmission, 420 (90.5 %) indicated abstinence as a prevention strategy (Table 2). However, 18.1 % believed that there exists a cure for HIV/AIDS and 3.4 % indicated sexual intercourse with a virgin as being curative.

Participants were also asked to name three formal sources of condom. Health centers, pharmacies, stores, outreach clinics, vending machines or any other formal structures or settings where condoms could be purchased or obtained free of charge made up the range of acceptable answers. Responses such as friends, family members, classmates, parents and teachers were informal sources and were not considered. Participants who could name all three were considered as having good knowledge, 2 correct as fair and only one correct as having poor knowledge. Only 48.3 % of participants had a good knowledge of sources of condom.

Relating to self-efficacy at the individual level, finding out the extent to which the participants felt capable of protecting themselves was crucial as this could enable them avoid unwanted sex and its consequences and demand condom use. They were thus asked if they could refuse sexual offers. This was to measure whether they felt confident that they had control over their sexual lives and activities. From the proposed responses, 50 % of the participants indicated they had very strong control over their sexual lives, 23.3 % had strong control, 6.9 % had no control while 6.0 % could not tell if they had any control.

Perception of peers’ sexual activity (peer norms) was another aspect considered in this study. Peer attitudes and norms could be an important influence on the behaviors of young people thus it was important to track attitudes and norms among young people because studies conducted both in developed and developing countries had demonstrated that, when adolescents believed their friends were engaging in sex, they were more likely to report having had sex themselves. Participants were thus asked about how many of their friends they thought had experienced sex. About 10.3 % of participants thought all of their friends had experienced sex, while 26.7 % reported none of their friends had had sex.

Adolescence is a very crucial phase in life and adolescents who perceive that their care givers support them have statistically higher levels of risk avoidance and lower levels of risk behaviors. HIV is ultimately driven by individual behavior; however, the context in which young people grow up and make decisions, including sexual decisions, contributes greatly to the types of decisions taken (i.e. whether to engage in risk behavior). Communication with primary caregivers was thus measured by asking participants how often their legal guardians talked to them about sex and safe practices. Only 37.1 % of the participants indicated that their legal guardian often talked to them about sex and safe practices, 41.4 % indicated they did sometimes while 20.7 % indicated that their legal guardians never talked to them.

With regards to knowledge-related questions, the overall mean (±SD) score of knowledge for all the participants was 12.91 (±1.925). Based on this, 62.1 % were qualified as having high level of knowledge with a knowledge score of ≥75 %, 34.3 % as medium level with 51 to 74 % score and 3.4 % as low level with ≤50 % score regarding HIV/AIDS.

### Attitudes toward PLHIV

Two hundred and forty four (52.6 %) respondents indicated a willingness to take care of a sick HIV-positive relative or continue friendship with an HIV-positive friend while only 56.9 % could buy food and other goods from an HIV-positive person (Table 3). The majority of the participants accepted that an HIV-positive student should be allowed to continue her/his studies (71.6 %) and that an HIV-positive teacher should be allowed to continue her/his teaching profession (75 %).

**Table 3:** Attitudes towards people living with HIV/AIDS

Question	Yes (%)	No (%)
1. If one of your relatives, who is HIV positive, becomes ill, would you be willing to care for her/him in your house or community?	244 (52.6)	216 (46.6)
2. If your friend is HIV positive, would you continue your friendship with him/her?	244 (52.6)	216 (46.6)
3. If a shopkeeper or food seller is HIV positive, would you buy items from him/her?	264 (56.9)	196 (42.2)
4. If a student is HIV positive, she/he should be allowed to continue his/her study in school?	332 (71.6)	128 (27.6)
5. If a teacher is HIV positive, she/he should be allowed to continue his/her teaching in school?	348 (75.0)	112 (24.1)

Only 52.5 % of students had positive attitudes towards PLHIV. Those who had negative attitudes comprised 47.5 %.

### Practices related to HIV/AIDS

As illustrated in Table 4, 196 (41.4 %) respondents had a history of sexual intercourse. The mean age (±SD) at sexual debut was 15.78 (±2.702) years. Of these, 124 (64.6 %) indicated that their last three sex partners were the same person, 108 (56.3 %) used a condom during their last three sexual encounters, 36 (18.8 %) were intoxicated during their last sexual encounter, and 36 (18.8 %) said their last sexual partner was at least 10 years older than them. One hundred and eighty eight (40.5 %) reported they had done an HIV test before, while only 42 (22.3 %) of these did the HIV test within 12 months prior to this study. Only 166 (38.1 %) knew their HIV status. None of the 464 participants used injectable drugs or share needles as a result.

**Table 4:** Practices related to HIV/AIDS

Variable	Yes (Number)	Yes (%)
Ever had sex before (*n* = 464)	196	41.4
Done HIV test before (*n* = 464)	188	40.5
Done HIV test past 12 months (*n* = 188)	42	22..3
Know HIV status (*n* = 42)	16	38.1
Use injectable drugs (*n* = 464)	00.0	00.00
If “Yes”, do you share needles with other drug users?	0.00	0.00
Last three sex partners was the same person (*n* = 196)	124	64.6
Used a condom during last 3 sexual intercourse (*n* = 196)	108	56.3
Intoxicated during their last sexual encounter (*n* = 196)	36	18.8
Last sexual partner was at least 10 years older (*n* = 196)	36	18.8

With a median score of 6, 59.4 % of the study participants reported safe practices, having a score equal to or greater than the median score from the questions on sexual practices/behavior regarding HIV/AIDS.

### Variation in KAP with respect to gender

KAP means and the standard deviations for both males and females were similar (Additional file 1). Considering the fact that more females (240) participated in the study than males (224), this unbalanced condition increased the susceptibility of both populations to unequal variances.

An independent samples t-test showed no significant difference in KAP (Additional file 1) between males and females (*t* = -1.425 (K), -1.062, 0.189 (A), df = 114, 114, 114 (P), *p* > .05 (KAP). Males (Mean = 12.64 (K), 2.93 (A), 2.84 (P), SD = 2.093 (K), 1.906 (A), 3.291 (P)) reported the same levels of KAP as females (Mean = 13.15 (K), 3.32 (A), 2.72 (P), SD = 1.735 (K), 2.021 (A), 3.664 (P)).

### Variation in KAP with respect to age of participants

Respondents were further categorized into the following age groups: 13–16 (*n* = 152), 17–20 (*n* = 280) and 21-24 (*n* = 32) and variation in KAP computed. Variations in KAP means across age groups were observed (Additional file 2). For knowledge and attitude, the variation was slight but large for behavior. Multivariate tests showed no significant differences in KAP between respondents of various age groups [*F* (6, 222) = 2.091, *p* > .05 (0.055); Wilk’s Λ = 0.896].

### Associations of knowledge with attitudes and practices

Bivariate correlations showed a positive, significant relationship between knowledge and attitude (*r* = +0.456, *P* = 0.000) (Additional file 3). This shows that respondents with medium and high levels of knowledge were likely to have proportionately positive attitudes than those with low level of knowledge.

A negative correlation was found to exist between knowledge and behaviors/practices but this was not significant (*r* = -0.014, *P* = 0.922) (Additional file 3).

## Discussion

Knowledge, attitudes and practices (KAP) studies are very useful tools prior to any intervention to assess the extent to which individuals or communities are ready to adopt risk-free behaviors [26]. In our study, 62.1 % of participants had a high level of knowledge of HIV/AIDS whereas those with poor knowledge comprised 3.4 %. Comparing with the report of Thanavanh et al. [27] in which 46.3 % had high levels of knowledge, and 22.4 %, poor knowledge, our participants were better informed about HIV/AIDS*.* Since the mid-1980s, extensive awareness campaigns on HIV/AIDS have been conducted locally, nationally and globally which could have been expected to have increased the HIV and AIDS knowledge of our participants. Similar results have been shown by Thanavanh et al*.* [27]. Similar to Haddison et al [22] our respondents reported sexual education in school as the main sources of information on HIV/AIDS. This implies that the school was a common source of HIV and AIDS information which augers well for school based HIV and AIDS programmes. With the exception of radio (16.4 %) the media (TV and newspapers) had the lowest ratings as sources of information. On the contrary, Bamise et al. [28] reported the media (Television, 76.9 %; radio, 75.5 %; newspapers/handbills, 74.4 %) as major sources of information on HIV/AIDS to secondary school adolescents in Nigeria. The low percentages of the media outlets as sources of information directed towards young people directly indicate that the media may not be the primary channel to use for any intervention on HIV/AIDS targeting adolescents in our study area*.* Young people usually turn to the media for entertainment rather than information [29].

We investigated knowledge of transmission and prevention of HIV infection on participants. Participants demonstrated awareness on routes of transmission of HIV/AIDS. Acceptable level of knowledge (excellent and good) was demonstrated by the majority of participants respectively. However misconceptions about transmission such as the believe that infection could be transmitted by mosquito bites (23 %), sex with an uninfected partner (15.5 %), non usage of condom (14.7 %), abstinence (9.5 %) and sharing a meal with an infected person (7.8 %) were observed among a small proportion of participants. These misconceptions could result in risky behaviors—unprotected sex or also multiple sexual partners etc, which may expose them to infection. These findings show the need for reinforcement of educational interventions particularly in the secondary school curriculum. Similar misconceptions have been reported by Mansoor [30], Koksal [31] and Tan [32]. In spite of these misconceptions, the majority of students were aware where to obtain condoms and indicated self-sufficiency in unwanted sex. Knowing a source of condoms is the first requisite for obtaining them but is not the same as actually being able to do so. Various barriers can prevent young people from accessing condoms, among the more common being their cost and the stigma associated with obtaining them. This notwithstanding, as illustrated on Table 4, an average usage of condom was observed (56.3 % of participants involved in sexual activity reported condom usage). This is similar to the 54.8 % reported by Andergie et al. [33] in Ethiopia. Condom usage in our study is however higher than 37 % reported by the 2011 DHS-MIC in Cameroon [34], indicating the effect of relentless efforts of sensitization.

Peer pressure is also another aspect influencing the behaviors of young people. To investigate this, participants were asked to estimate the proportion of their friends they thought were engaging in sexual activities. Only 26.7 % indicated that none of their friends were engaging in sexual activities. However, since most of the studies which have analyzed the influence of perception of friends’ sexual behavior on that of an individual have been longitudinal [35], it is still not clear whether the relationship is causal. For example, it could be that adolescents mimic the actual or imagined behavior of their peers, or it could be that once adolescents initiate sexual activity they are more likely to assume that their peers are also sexually active than would otherwise be the case. If the former be the case, peer norms could be a risk factor, possibly contributing to early sexual activity. However, peer norms can also have a positive effect because adolescents who believe that their peers are using condoms might be more likely to use condom and those who believe their peers are abstaining might be more likely to abstain.

With respect to PLHIV, students exhibited mixed attitudes displaying positive attitudes on some of the issues and negative attitudes on others. Overall, only 52.5 % of participants had a positive attitude towards PLHIV. This shows that discriminatory attitudes were present in a considerable proportion (47.5 %) of the participants. However, a greater majority accepted that HIV positive students could be allowed to continue their education (71.6 %) and that an infected teacher should continue teaching (78.0 %). Discriminatory attitudes towards PLHIV might be an obstacle for the efficient propagation of awareness programs, and voluntary counseling and testing for HIV. A significant positive correlation (*r* = +0.456, *P* = 0.000) was observed between knowledge and attitude (Additional file 3) showing that good knowledge could contribute to positive attitude as it could mean a better understanding of transmission. Thus sustained education of young people on HIV/AIDS is crucial to the elimination of discriminatory attitudes towards PLHIV.

Regarding practices, 41.4 % of the participants had been engaged in sexual activity with mean age of sexual debut being 15. 78 years. This is similar to 16 years reported in South Africa [36] as age at sexual debut. However, studies in other African countries [37] have reported an earlier age for first sexual encounter. The proportion of sexually active students reported in our study is lower than 43.7 % reported by Haddison et al*.* [22] and 54 % reported by Tarkang [23] but higher than 14.9 % reported by Andargie *at al*. [33] in Ethiopia and Thanavanh et al. [27] in Lao People’s Republic. Sex is now viewed as a norm among young people and this might explain the early age at which they start engaging in sexual activities. Only 56.3 % indicated that they had used condom during their last three sexual encounters. However, condom use at last sex provides no measure of the consistency of condom use. Increases in the rate of condom use at last sex, therefore, while a positive sign, do not mean that the young people reporting condom use have not placed themselves at risk of acquiring HIV infection at any time in the preceding 12 months. Of the 196 (41.4 %) participants with reported sexual activity, 64.6 % indicated that their last three sexual encounters were with the same person while 35.5 % reported having different partners. Compared to the 2011 DHS-MIC survey report [34] in which 6 and 29 % of females and males respectively had multiple sexual partners, a considerable number of our study participants had the tendency of having multiple sexual partners. Age-mixing in sexual partners among young people was also noted in our study as 18.8 % of sexually active participants reported that their last sexual partner was at least 10 years older than them. This is a very risky practice especially for young women as this has been reported to women’s risk of infection [38, 39]. Sex between young women and older men is often more risky because in such relationships young women lack the power to negotiate safe sex. It is also an efficient means of spreading HIV infection, since, for physiological and anatomical reasons younger women are more likely to become infected. Each sexual act with an infected man carries a higher risk of infection for a young girl. Sex between younger women and older men is socially unacceptable. Engaging in sex while intoxicated, another risky practice was reported by 18.7 % of the participants. Sexual intercourse, while one or both partners are intoxicated, is more likely to be unplanned, and couples are therefore less likely to use condoms.

HIV testing was poor among students as only 40.5 % had done an HIV test and 22.3 % (42/188) of these did the test within 12 months prior to our study. Although voluntary counseling and testing (VCT) was generally poor among study participants (40.5 %), our proportion of students who had tested for HIV is greater than the 28.7 % reported by Haddison et al. [22]. Among those did the test, only 38.1 % knew their HIV status. These are individuals who collected their results. The rest never collected for their results as such could not know their status. Haddison et al. [22] reported similar findings. Factors that could have contributed to this result were cost, perception of the confidentiality of the process, and especially, of the result, and the perceived attitude of the staff towards young people.

The use of injectable drugs was not seen in this study and thus the consequent risky practice of exchanging syringes/needles was absent. The use of injectable drugs is not a common practice among youths in Cameroon. A negative correlation was observed between knowledge and practices on HIV/AIDS but this was not statistically significant (-0.014, *P* = 0.922) (Additional file 3). Ankomah et al*.* [40] reported similar findings though their study was on adult men.

There were no significant differences in KAP between male and female participants. Significant differences in KAP were also not observed between participants in various age groups. HIV/AIDS-related activities should be focused on adolescents because this is the age when sexual activity begins. At this age most adolescents are in school thus are accessible through in-school education. In our findings, this was confirmed as the most effective way of reaching students because it was picked as the main source of information on HIV/AIDS by 66.4 % of the participants. Accurate HIV knowledge will support adolescents in making informed choices about practices that may protect them from HIV infection.

## Conclusions

The level of knowledge regarding HIV/AIDS transmission, prevention and control was considered satisfactory. However, some misconceptions about HIV transmission, risky behaviors and discriminatory attitudes were observed among participants that call for concern and must be addressed promptly. Sexual education in schools, should be reinforced to correct the misconceptions observed in this study and encourage safe practices and positive attitudes towards PLHIV.

### Limitations of the study

We restricted this study to only one Division in the South West Region and all the participating institutions were day schools. This therefore limits the generalizability of the study findings to other regions and possibly to boarding secondary schools. Although HIV knowledge is very important, it may not necessarily be the primary factor in explaining HIV transmission among young people. Many young people have adequate knowledge about HIV but fail to act on it due to a wide variety of social, cultural and economic constraints or just out of pure negligence. Future studies that investigate all these possible constraints could greatly help to improve our understanding of HIV transmission especially among young people. Finally, because the questionnaire was self-administered, social desirability bias may have occurred. However, the anonymity of the questionnaires hopefully encouraged students to be honest in their responses. Despite all of these limitations, we believe this study might be a reasonable source of information for researchers and policymakers.

## Additional files

Additional file 1: Variation in KAP by gender. This shows an analysis of variation in KAP between male and female participants. (XLSX 15 kb) Additional file 2: Variation in KAP by age group of participants. Description of data: This presents a comparative analysis of KAP between study participants in various age groups. (XLSX 14 kb) Additional file 3: Correlation between knowledge and attitude, and knowledge and practice. This shows the relationship of participants’ knowledge on HIV/AIDS and their attitude towards people living with HIV and also the relationship of their knowledge and their practices on HIV prevention. (XLSX 11 kb)

## References

[1] World Health Organization. Global Health Observatory Data. HIV/AIDS.www.who.int/gho/hiv/en. Accessed 28 Nov 2015.

[2] UNICEF. HIV/AIDS Global and Regional Trends. http://data.unicef.org/hiv-aids/global-trends. Accessed 9 Apr 2015.

[3] Joint United Nations Program on HIV/AIDS. At the Crossroads: Accelerating Youth Access to HIV/AIDS Interventions. www.un.org/esa/socdev/unyin/documents/aidsunfpa.pdf Accessed 28 Apr 2014.

[4] Chen FP (2008). HIV/AIDS prevent among young people in East and South-East Asia in the context of reproductive and sexual health. Asia Pac Popul J.

[5] UNAIDS. Beginning of the end of the AIDS epidemic. The Gap Report. www.unaids.org/sites/default/files/media_asset/UNAIDS_Gap_report_en.pdf. Accessed 28 May 2015.

[6] Sentinel (2000). Surveillance of the Ministry of Public Health Cameroon.

[7] National AIDS Control Committee Central Technical Group. The Impact of HIV and AIDS in Cameroon through 2020. 40pp. www.healthpolicyinitiative.com/Publications/Documents/1250_1_Cameroon_EN_Singles_Reduced_acc.pdf.

[8] UNAIDS. HIV and AIDS estimates 2013. http://www.unaids.org/en/regionscountries/countries/cameroon. Accessed 25 Apr 2014.

[9] Mbanya D, Sama M, Tchunwou P (2008). Current status of HIV/AIDS in Cameroon: How effective are control strategies?. Int J Environ Res Public Health.

[10] UNAIDS. Report on the Global AIDs Epidemic. www.unaids.org/sites/default/files/media_asset/UNAIDS_Global_Report_2013_en_1.pdf. Accessed 15 Apr 2015.

[11] The Demographic Health Survey Program. Cameroon DHS, 2011-HIV Fact Sheet. www.dhsprogram.com/publications/publication-HF42-HIV-Fact-Sheets.cfm. Accessed 22 Apr 2015.

[12] WHO. Cameroon: Summary of Country Profile for HIV/AIDS treatment scale-up. www.who.int/hiv/HIVCP_CMR.pdf. Accessed 15 Apr 2015.

[13] Plautz A, Meekers D (2007). Evaluation of the reach and impact of the 100 % Jeune youth social marketing program in Cameroon: findings from three cross-sectional surveys. Reprod Health.

[14] Lydie N, Robinson NJ, Ferry B, Akam E, De Loenzien M, Zekeng L, Abega S (2004). Adolescent sexuality and the HIV epidemic in Yaounde, Cameroon. J Biosoc Sci.

[15] Herek GM, Capitanio JP, Widaman KF (2002). HIV – related stigma and knowledge in the United States: prevalence and trends, 1991–1999. Am J Public Health.

[16] Dimbuene ZT, Defo BK (2011). Fostering accurate HIV/AIDS knowledge among unmarried youths in Cameroon: Do family environment and peers matter?. BMC Public Health.

[17] Tarkang EE. HIV knowledge and its association with sexual behaviours among out – of – school adolescents in Kumba, South West Region of Cameroon. Int STD Res and Rev. 2014a; 2(2): 123-134

[18] Rwenge M (2000). Sexual risk behavious among young people in Bamenda, Cameroon. Int Fam Plann Persp.

[19] Meekers D, Klein M, Foyet L (2003). Patterns of HIV risk behaviour and condom use among youth in Yaounde and Douala, Cameroon. AIDS Behav.

[20] Meekers D, Klein M (2003). Determinants of condom use among young people in urban Cameroon. Stud Fam Plann.

[21] Tarkang EE. Factors associated with perception of risk of contracting HIV among secondary school female learner in Mbonge subdivision of rural Cameroon. Pan Afr Med J. 2014b; 17: 259 10.11604/pamj.2014.17.259.2772 PMC4189865 25309659

[22] Haddison EC, Nguefack – Tsague G, Noubom M, Mbacham W, Ndumbe PM, Mbopi-Keou FX (2012). Voluntary Counseling and testing for HIV among high school students in Tiko health district, Cameroon. Pan Afr M J.

[23] Tarkang EE. Age at sexual debut and associated factors among high school female learner in Limbe urban area of Cameroon. Glob Adv Res J Soc Sci. 2013a; 2(7): 163 – 168

[24] Tarkang EE. Correlates of consistent condom use among secondary school female students in Limbe urban city, Cameroon. Int. J. Curr. Microbiol. App Sci. 2013b; 2(8): 245 – 259

[25] WHO. National AIDS programmes: a guide to indicators for monitoring and evaluating national HIV/AIDS prevention programmes for young people. www.who.int/maternal_child_adolescent/documents/9241592575/en/. Accessed 5 Mar 2014.

[26] National AIDS Control Committee Central technical Group. The impact of HIV and AIDS in Cameroon through 2020. www.healthpolicyinitiative.com/Publications/Documents/1250_1_Cameroon_EN_Singles_Reduced_acc.pdf…. Accessed 22 Apr 2015.

[27] Thanavanh B, Rashid HO, Kasuya H, Sakamoto J (2013). Knowledge, attitudes and practices regarding HIV/AIDS among male high school students in Lao Peoples Democratic Republic. J Int AIDS Soc.

[28] Bamise OF, Bamise CT, Adedigba MA (2011). Knowledge of HIV/AIDS among secondary school adolescents in Osun State, Nigeria. Niger J Clin Pract.

[29] https://www.commonsensemedia.org/research/children-teens-and-entertainment-media-the-view-from-the-classroom. Children, Teens, and Entertainment Media. The view from the classroom. Accessed 4 Nov 2015.

[30] Mansoor AB, Fungladda W, Kaewkungwal J, Wongwit W (2008). Gender differences in KAP related to HIV/AIDS among freshmen in Afghan universities. Southeast Asian J Trop Med Public Heath.

[31] Koksal S, Narmal N, Vehid S, Yurtsever E (2005). Knowledge and attitude towards HIV/AIDS among Turkish students. Infecti Dis J Pak.

[32] Tan X, Pan J, Zhou D, Wang C, Xie C (2007). HIV AIDS Kunled, attitudes and behaviours assessment of Chinese students: questionnaire study. Int J Environ Res Public Health.

[33] Andargie G, Kassu A, Moges F, Kebede Y, Gedefaw M, Wale F, Alem A, Andualem B, Adungna S (2007). Low prevalence of HIV infection, and knowledge, attitude and practice on HIV/AIDS among high school students in Gondar, Northwest Ethiop. J Health Dev.

[34] National Institute of Statistics (2012). The 2011 Cameroon Demographic and Health Survey and Multiple Indicators Cluster Survey (DHS-MICS 2011). www.statistics-cameroon.org/downloads/EDS-MICS11/DHSMIC_2011_preliminary_report.pdf. Accessed 4 Nov 2015.

[35] Sieving RE, Eisenberg ME, Pettingell S, Skay C (2006). Friends influence adolescents’ first sexual intercourse. Perspect Sex Reprod Health.

[36] Zuma K, Setswe G, Ketye T, Mzolo T, Mbelle N (2010). Age at sexual debut: a determinant of multiple partnership among South African Youth. Afr J Reprod Health.

[37] Mmbaga JE, Leonard F, Leyna GH (2013). Incidence and predictors of adolescent’s early sexual debut after 3 decades of HIV interventions in Tanzania: A time to debut analysis. PLoS One.

[38] Sa Z, Larsen U (2008). Gender inequality increases women’s risk of HIV infection inMoshi, Tanzania. J Bioscoc Sci.

[39] Kelly RJ, Gray RH, Sewankambo NK, Serwadda D, Wabwire-Mangen F, Lutalo T, Wawer MJ (2003). Age differences in sexual partners and risk of HIV-1 infection in rural Uganda. JAcquir Immune Defic Syndr.

[40] Ankomah A, Adebayo SB, Anyanti J, Ladipo O, Ekweremadu B (2013). Determinants of condom use by men in extramarital relationships in Nigeria. HIV/AIDS Res Palliative Care.

